# Supplementary material for: Modulation of Neuronal Excitability and Plasticity by BHLHE41 Conveys Lithium Non-Responsiveness
Source: bioRxiv. 2024 Jul 25:2024.07.25.605130. Preprint. [Version 1] doi: 10.1101/2024.07.25.605130 (PMC11451663; doi:10.1101/2024.07.25.605130)
Supplement: Supplement 1 [file NIHPP2024.07.25.605130v1-supplement-1.pdf]

bioRxiv preprint doi: https://doi.org/10.1101/2024.07.25.605130; this version posted July 25, 2024. The copyright holder for this preprint (which was not certified by peer review) is the author/funder, who has granted bioRxiv a license to display the preprint in perpetuity. It is made available under aCC-BY 4.0 International license.

Supplementary Figures

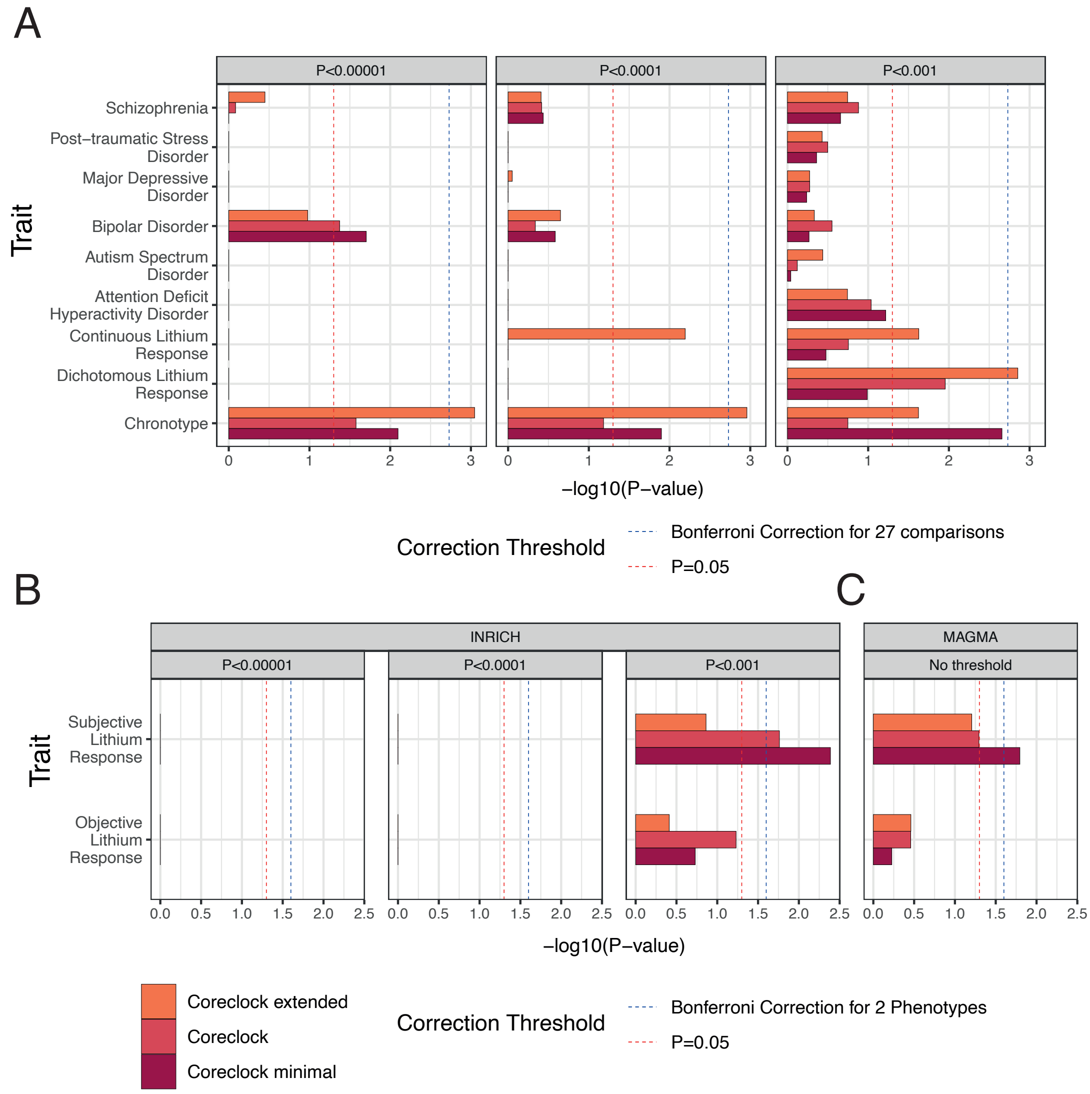

Figure S1. INRICH analyses based on core clock gene-sets in psychiatric traits. (A) INRICH analyses of data shown in Figure 1. Bonferroni significance thresholds: dashed blue line (P < 0.05/ (3 gene-sets x 9 traits)). Three different P-value thresholds were analyzed (P=0.00001, P=0.0001, P=0.001). The ‘Coreclock extended’ gene-set reaches corrected p-val-threshold at P=0.00001 and P=0.0001 in the Chronotype sample, whereas it reaches corrected p-val-threshold in the ‘Dichotomous Lithium Response’ sample at P=0.001. (B) INRICH analyses and (C) MAGMA enrichment analyses of swedish replication cohort with two phenotypes: subjective and objective lithium response. Bonferroni significance thresholds: dashed blue line (P < 0.05/ (3 gene-sets x 2 traits)). (Competitive test P-values in the X-axis are –log10 converted. Dashed red line indicates the nominal (uncorrected) significance threshold P=0.05.

bioRxiv preprint doi: https://doi.org/10.1101/2024.07.25.605130; this version posted July 25, 2024. The copyright holder for this preprint (which was not certified by peer review) is the author/funder, who has granted bioRxiv a license to display the preprint in perpetuity. It is made available under aCC-BY 4.0 International license.

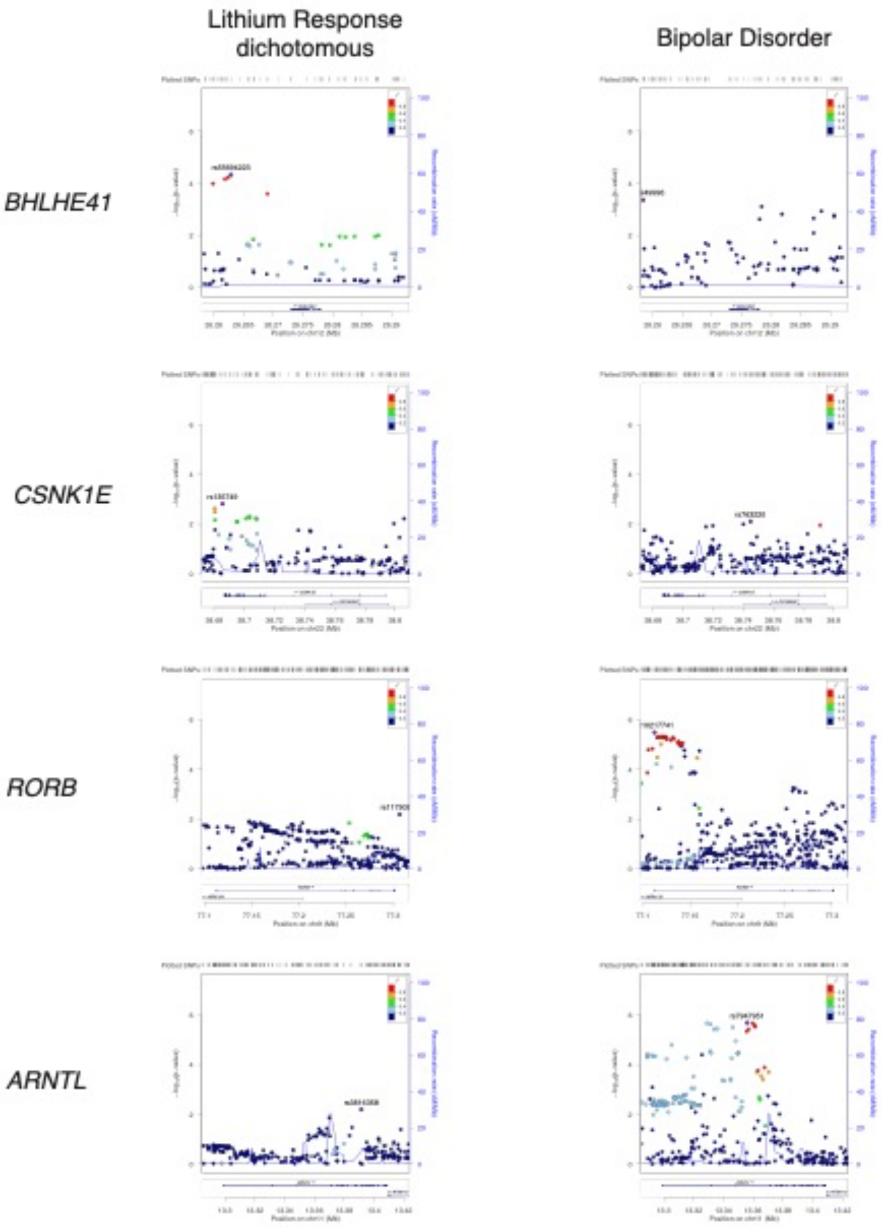

Figure S2. Manhattan plots for nominally significant contributing genes in the core clock gene-set and ARNTL. Manhattan plots from GWAS data on dichotomous lithium response (A, C, E, G) and bipolar disorder risk (B, D, F, H). For the top three (and nominally significant) lithium response-associated genes from the core clock gene-set: (A, B) *BHLHE41*, (C, D) *CSNK1E*, and (E, F) *RORB*. In addition, the same diagrams are shown for *ARNTL* (G, H), a GWAS associated BD risk gene also known as the core clock regulator *BMAL1*.

bioRxiv preprint doi: https://doi.org/10.1101/2024.07.25.605130; this version posted July 25, 2024. The copyright holder for this preprint (which was not certified by peer review) is the author/funder, who has granted bioRxiv a license to display the preprint in perpetuity. It is made available under aCC-BY 4.0 International license.

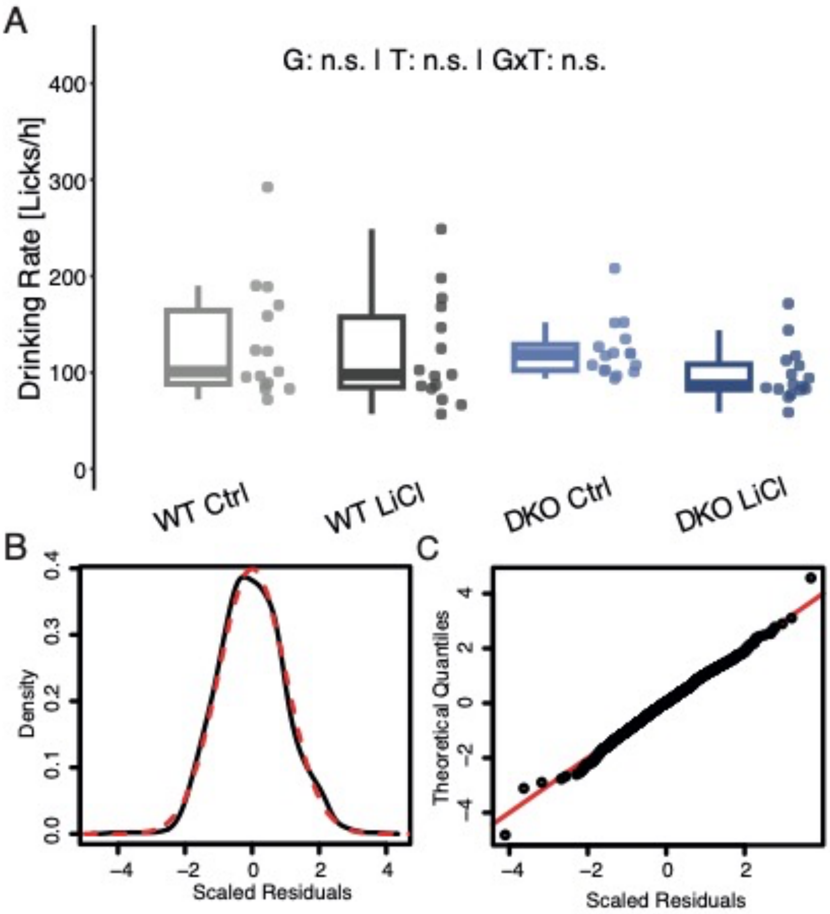

Figure S3. Quality controls for the PsyCoP behavioral study. (A) Drinking rate in the IntelliCage during the experiments was not significantly different in lithium chloride-treated mice compared to control. Data are shown as box plots with whiskers extending to no more than 1.5-fold IQR. (B) Density plot and QQ plot of the scaled residuals (black) of the multivariate linear model used for the ANOVA procedure of all PsyCoP measures compared to a normal distribution (red) show no major deviations. * P < 0.05, ** P < 0.01, *** P < 0.001, n.s. not significant; P-values are FDR-adjusted and refer to Wilk's lambda testing two-way ANOVA; G: genotype term; T: lithium treatment term; GxT: interaction term; WT: wildtype; DKO: Bhlhe40/41 double-knockout; Ctrl: vehicle control; LiCl: lithium-treated

bioRxiv preprint doi: https://doi.org/10.1101/2024.07.25.605130; this version posted July 25, 2024. The copyright holder for this preprint (which was not certified by peer review) is the author/funder, who has granted bioRxiv a license to display the preprint in perpetuity. It is made available under aCC-BY 4.0 International license.

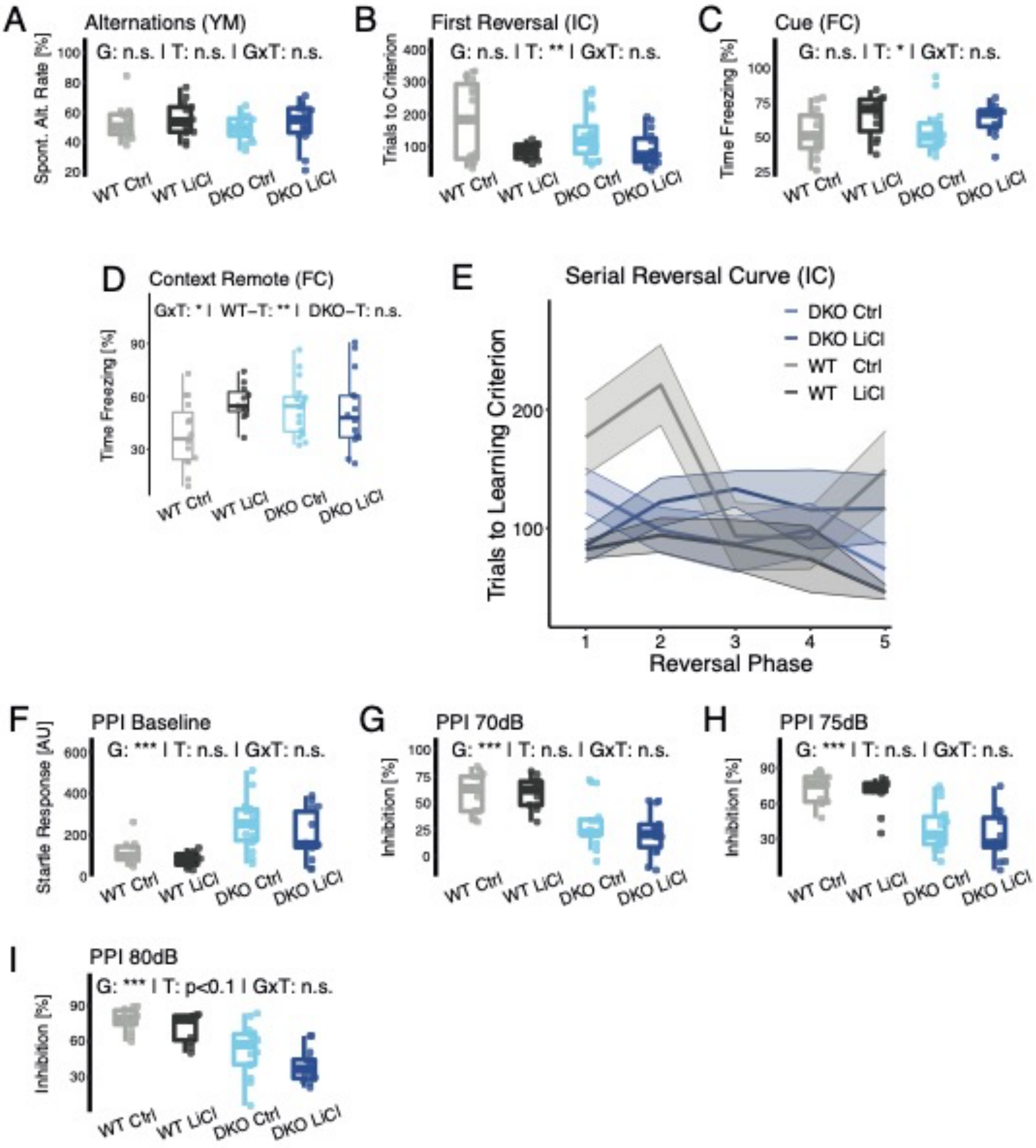

Figure S4. Remaining variables of the cognition and sensorimotor RDoC domains. (A-C) display the variables of the Cognitive RDoC domain not shown in Figure 2. (A) There was no significant effect on working memory in the Y-Maze test measured with the rate of spontaneous alternations between arms. (B) In the first reversal lithium treated mice needed less trials to reach the learning criterion indicating improved learning flexibility. Although, the difference in DKO mice was smaller than in wildtypes, the interaction was not significant in this part of the spatial learning experiments. (C) Similarly, in the cued fear memory task, lithium treatment increased the time spent freezing, showing improved cued fear memory, with no significant influence in DKOs. (D) In the remote contextual fear memory, however, there was a significant Li-treatment dependent effect between the genotypes, as indicated. (E) The ribbon plot of this curve refers to the Serial Reversal Task analysis (Fig. 2E) and displays the mean number of trials each group needed to reach the spatial learning criterion (10% better success rate than random expectation) after each reversal of the correct corner. Smaller values equal less trials needed and indicate faster learning success. WT animals improve upon Lithium treatment, whereas DKO performance remains unaltered. (F-I) show the variables of the Sensorimotor domain with a strong genotype effect in all of them, but only minor influence of lithium treatment on the highest prepulse level (PPI 80dB) in panel (I). Data are shown as box plots with whiskers extending to no more than 1.5-fold IQR; * P < 0.05, ** P < 0.01, *** P < 0.001, n.s. not significant; P-values are FDR-adjusted and refer to Wilk's lambda testing two-way ANOVA; G: genotype term; T: lithium treatment term; GxT: interaction term; WT: wildtype; DKO: Bhlhe40/41 double-knockout; Ctrl: Placebo control; LiCl: lithium-treated

bioRxiv preprint doi: https://doi.org/10.1101/2024.07.25.605130; this version posted July 25, 2024. The copyright holder for this preprint (which was not certified by peer review) is the author/funder, who has granted bioRxiv a license to display the preprint in perpetuity. It is made available under aCC-BY 4.0 International license.

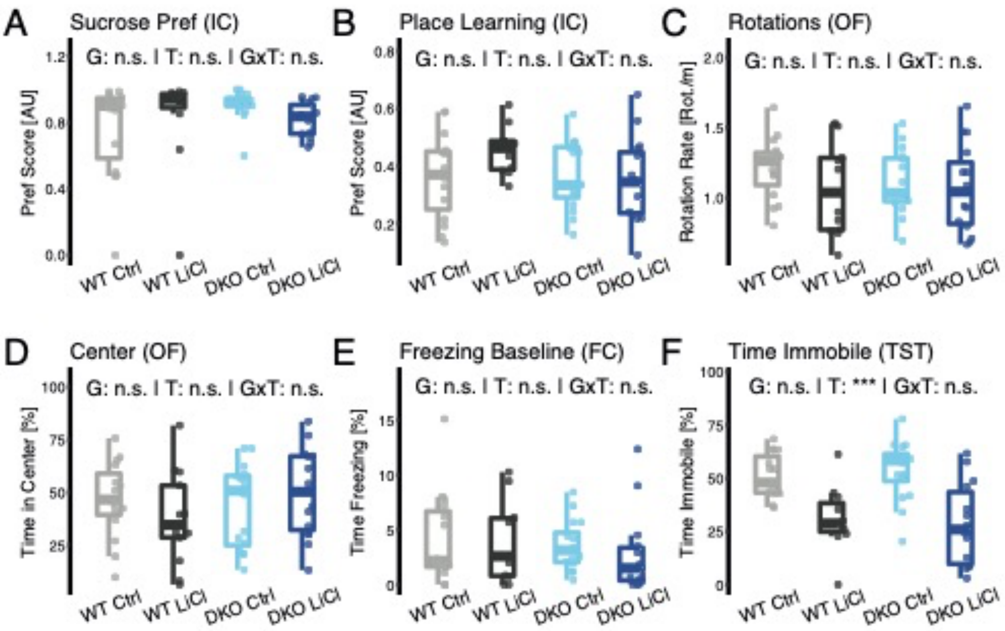

Figure S5. Positive and Negative Valence RDoC domains. The variables associated to positive valence systems did not show significant effects, neither in the Sucrose Preference Test (A) nor the place preference in Positive Reinforcement Learning (B). Notably, there was sex difference in sucrose preference detected, shown in Figure S7. In the negative valence domain parameters, there were no significant differences in rotation rate (C) and the center time (D) in the Open Field Test as well as the baseline freezing behavior (E) in Fear Conditioning either. However, lithium-treated mice were significantly less immobile (F) in the Tail Suspension Test independent of genotype. Data are shown as box plots with whiskers extending to no more than 1.5-fold IQR; * P < 0.05, ** P < 0.01, *** P < 0.001, n.s. not significant; P-values are FDR-adjusted and refer to Wilk's lambda testing two-way ANOVA; G: genotype term; T: lithium treatment term; GxT: interaction term; WT: wildtype; DKO: Bhlhe40/41 double-knockout; Ctrl: Placebo control; LiCl: lithium-treated

bioRxiv preprint doi: https://doi.org/10.1101/2024.07.25.605130; this version posted July 25, 2024. The copyright holder for this preprint (which was not certified by peer review) is the author/funder, who has granted bioRxiv a license to display the preprint in perpetuity. It is made available under aCC-BY 4.0 International license.

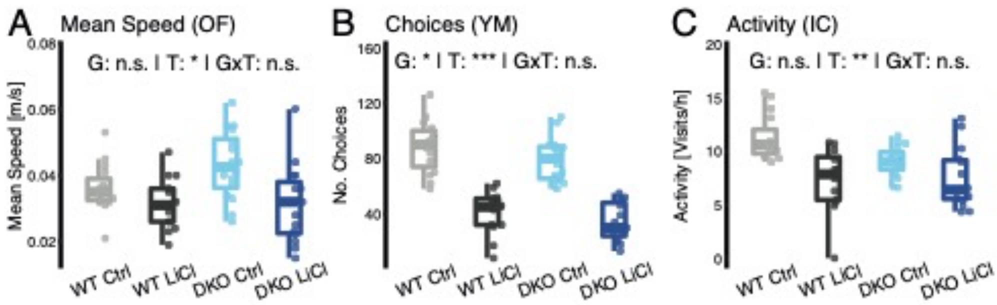

Figure S6. Arousal and Regulatory RDoC domain. Novelty-induced activity was reduced in lithium-treated animals (A) measured as Mean Speed in the Open Field Test and (B) the number of arm choices in the Y-Maze Test. In latter case Bhlhe40/41 DKO mice displayed a slightly reduced activity, as well, independent of treatment. General Activity (C) in the IntelliCage was similarly reduced in response to lithium treatment. Please note that there was a sex difference in activity level in response to lithium treatment shown in Figure S7 with males being impacted stronger than females. Data are shown as box plots with whiskers extending to no more than 1.5-fold IQR; * P < 0.05, ** P < 0.01, *** P < 0.001, n.s. not significant; P-values are FDR-adjusted and refer to Wilk’s lambda testing two-way ANOVA. G: genotype term; T: lithium treatment term; GxT: interaction term; WT: wildtype; DKO: Bhlhe40/41 double-knockout; Ctrl: Placebo control; LiCl: lithium-treated

bioRxiv preprint doi: https://doi.org/10.1101/2024.07.25.605130; this version posted July 25, 2024. The copyright holder for this preprint (which was not certified by peer review) is the author/funder, who has granted bioRxiv a license to display the preprint in perpetuity. It is made available under aCC-BY 4.0 International license.

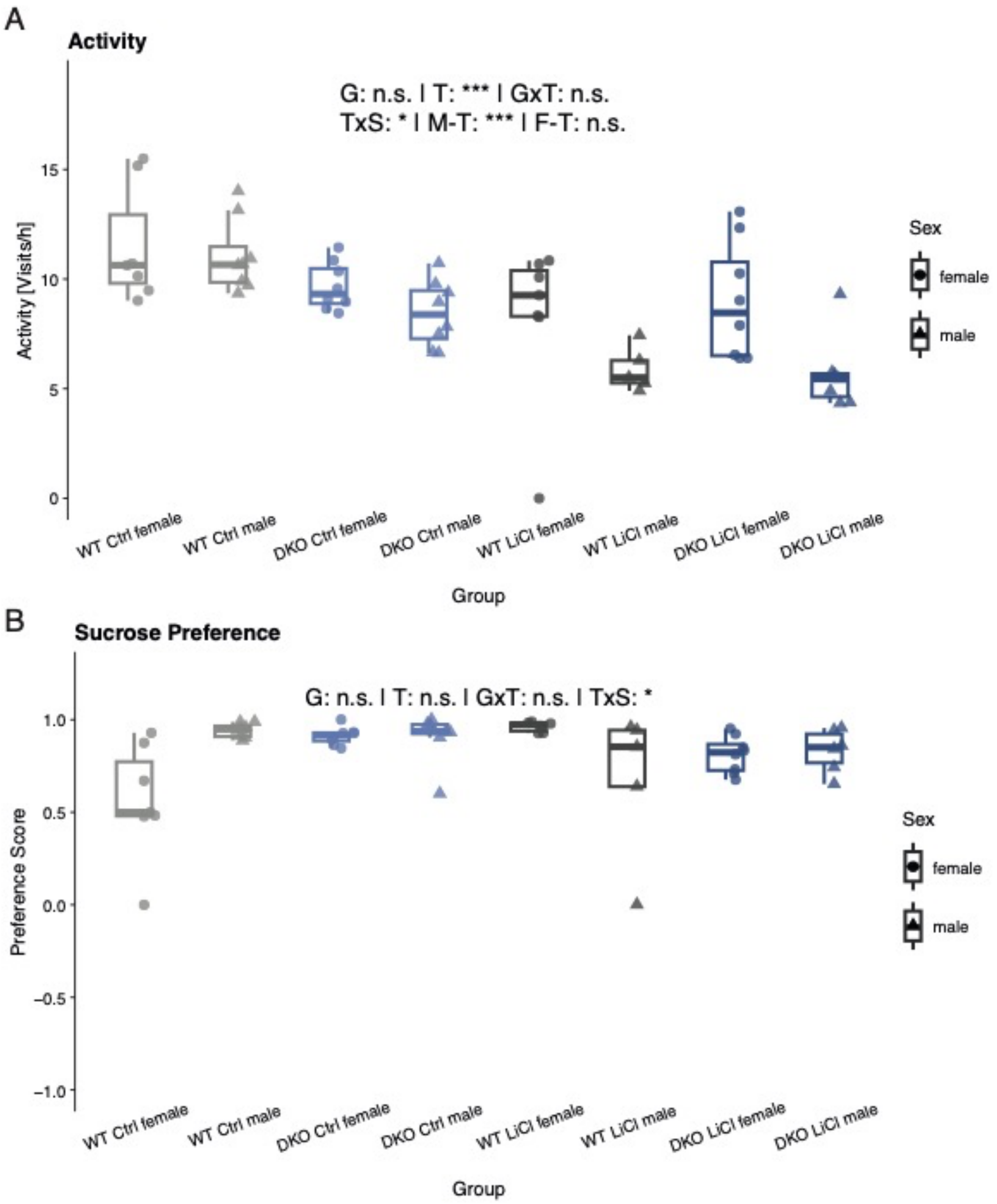

Figure S7. Sex Differences in general activity and sucrose preference. In most behavioral parameters assessed, we did not detect significant differences between sexes (Table S3). (A) However, male mice showed a stronger reduction in general activity in response to lithium treatment than female mice. (B) Moreover, the female wildtype placebo control (WT Ctrl female) group displayed a lower sucrose preference than their lithium-treated counterpart. This difference in lithium response between sexes was not found in DKO mice. Data are shown as box plots with whiskers extending to no more than 1.5-fold IQR; * $P < 0.05$, ** $P < 0.01$, *** $P < 0.001$, n.s. not significant; P-values are FDR-adjusted and refer to Wilk's lambda testing two-way ANOVA; n = ; G: genotype term; T: lithium treatment term; GxT: G by T interaction term; TxS: T by sex interaction term; M-T: male treatment effect; F-T: female treatment effect; WT: wildtype; DKO: Bhlhe40/41 double-knockout; Ctrl: Placebo control; LiCl: lithium-treated

bioRxiv preprint doi: https://doi.org/10.1101/2024.07.25.605130; this version posted July 25, 2024. The copyright holder for this preprint (which was not certified by peer review) is the author/funder, who has granted bioRxiv a license to display the preprint in perpetuity. It is made available under aCC-BY 4.0 International license.

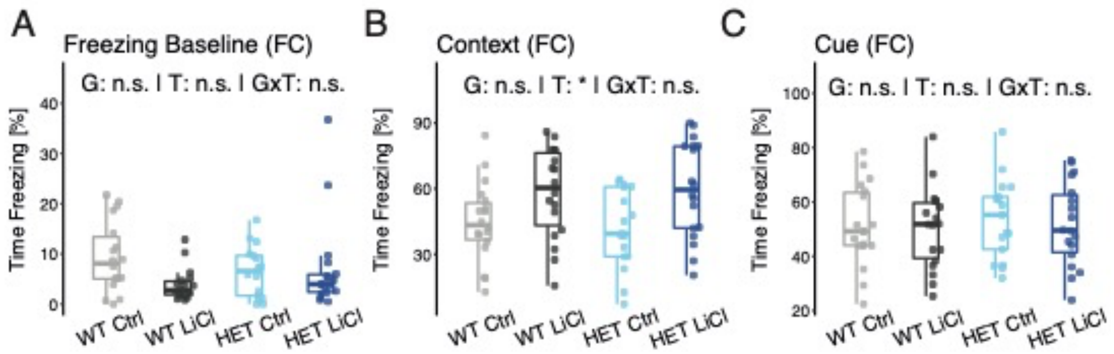

Figure S8. Fear Conditioning of Arntl mouse model. (A-C) Arntl heterozygous null mutant mice (Het) and littermate controls untreated or treated with lithium were subjected to fear conditioning (FC) paradigm. (A) No differences were detected at baseline freezing. (B) Testing contextual fear revealed a treatment dependent significant increase in both genotypes. (C) Cued FC detected no changes. Data are shown as box plots with whiskers extending to no more than 1.5-fold IQR; * P < 0.05, ** P < 0.01, *** P < 0.001, n.s. not significant; P-values are FDR-adjusted and refer to Wilk's lambda testing two-way ANOVA; n = ; G: genotype term; T: lithium treatment term; GxT: G by T interaction term; TxS: T by sex interaction term; M-T: male treatment effect; F-T: female treatment effect; WT: wildtype; Het: Arntl heterozygous null mutants; Ctrl: Placebo control; LiCl: lithium-treated

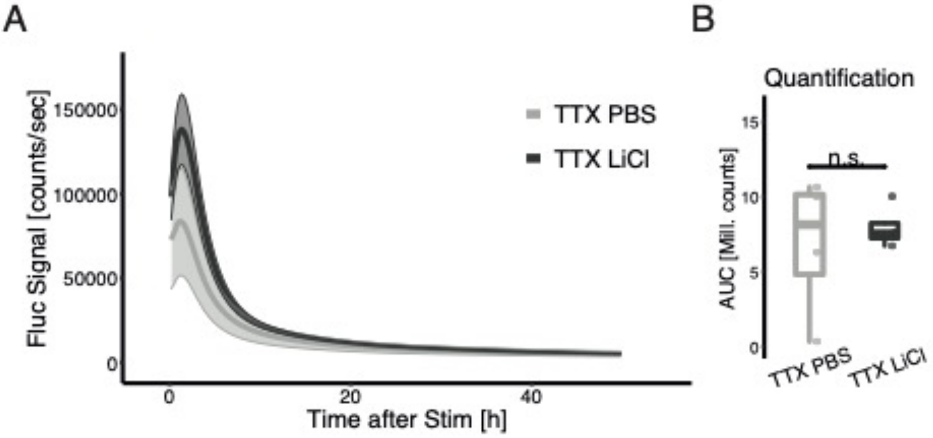

Figure S9. Lithium toxicity. (A) First 50 hours of tetrodotoxin (TTX)- and AP-V-silenced primary cortical neuron culture from wildtype (WT) embryos after lithium treatment. Cells were transduced with an ESARE-Firefly Luciferase (Fluc) reporter construct via AAV infection. After 7 days of pretreatment with lithium chloride (LiCl), a TTX/AP-V cocktail was added to silence neuronal network activity. At the start of the recording a typical artifact peak resulting from the time-lag of the transcriptional-translational reporter system, was seen. (B) The area under the curve of the first 100 hours of recording was quantified. No statistically significant difference was found in a Student's two-sided t-test, indicating that differences in ESARE-response are due to activity dampening not cytotoxic effects of lithium chloride. n.s.: not significant.

bioRxiv preprint doi: https://doi.org/10.1101/2024.07.25.605130; this version posted July 25, 2024. The copyright holder for this preprint (which was not certified by peer review) is the author/funder, who has granted bioRxiv a license to display the preprint in perpetuity. It is made available under aCC-BY 4.0 International license.

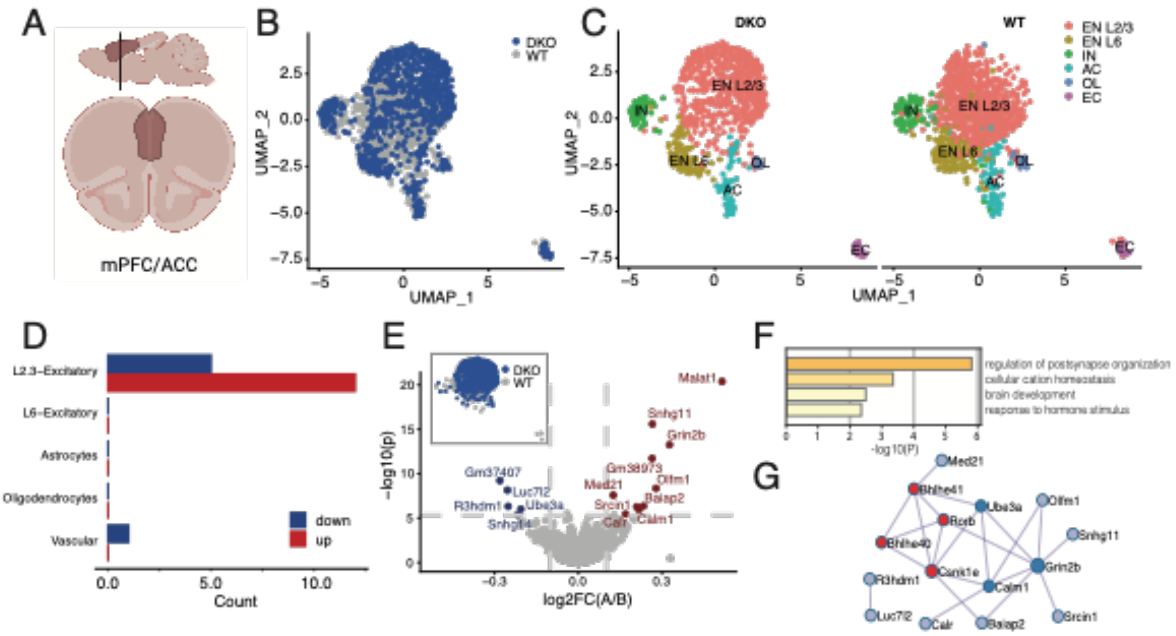

Figure S10. Layer 2/3 excitatory neurons in the mPFC of BHLHE40$^{-/-}$/41$^{-/-}$ mice show transcriptional changes in genes associated with the post-synapse. (A) The anterior cingulate cortex (ACC) part of the medial prefrontal cortex (mPFC) was selected as region of interest and isolated at Zeitgeber Time (ZT) 4, at circadian trough of BHLHE41 expression for single-nucleus RNA sequencing (snRNAseq) to identify constitutively deregulated genes. (B) UMAP dimension plot of the integrated datasets from wildtype (WT) and BHLHE40$^{-/-}$/41$^{-/-}$ (DKO) shows successful integration. (C) Six major cell populations, shown in distinct colors, were identified in both WT and DKO tissue samples. (D) Barplot of numbers of significantly deregulated genes between the genotypes in all cell clusters reveals a selective response in layer 2/3 cells only. (E) A Differential expression analysis between WT and DKO Layer 2/3 excitatory neurons (EN L2/3) identified a small set of differentially expressed genes. The gray lines indicates a threshold of 0.1 average log2-transformed fold-change (log2FC) and the Sidak-corrected significance threshold of the -log10-transformed p-value, assuming independent tests. (F) Gene ontology clusters enriched in the differentially expressed gene (DEG) set find with MetaScape[1] hint to a postsynapse-related mechanism. (G) The top genes identified in the human GWGAS (red) form a protein-protein interaction (PPI) network with the DEGs identified in ACC-derived EN L2/3 (blue) with Grin2b, Calm1, and Ube3a (dark blue) at its center. EN L2/3: excitatory neurons layer 2/3 (upper layer); EN L6: excitatory neurons layer 6 (deeper layer); IN: inhibitory neurons; AC: astrocytes; OL: oligodendroglial cells; EC: endothelial cells

bioRxiv preprint doi: https://doi.org/10.1101/2024.07.25.605130; this version posted July 25, 2024. The copyright holder for this preprint (which was not certified by peer review) is the author/funder, who has granted bioRxiv a license to display the preprint in perpetuity. It is made available under aCC-BY 4.0 International license.

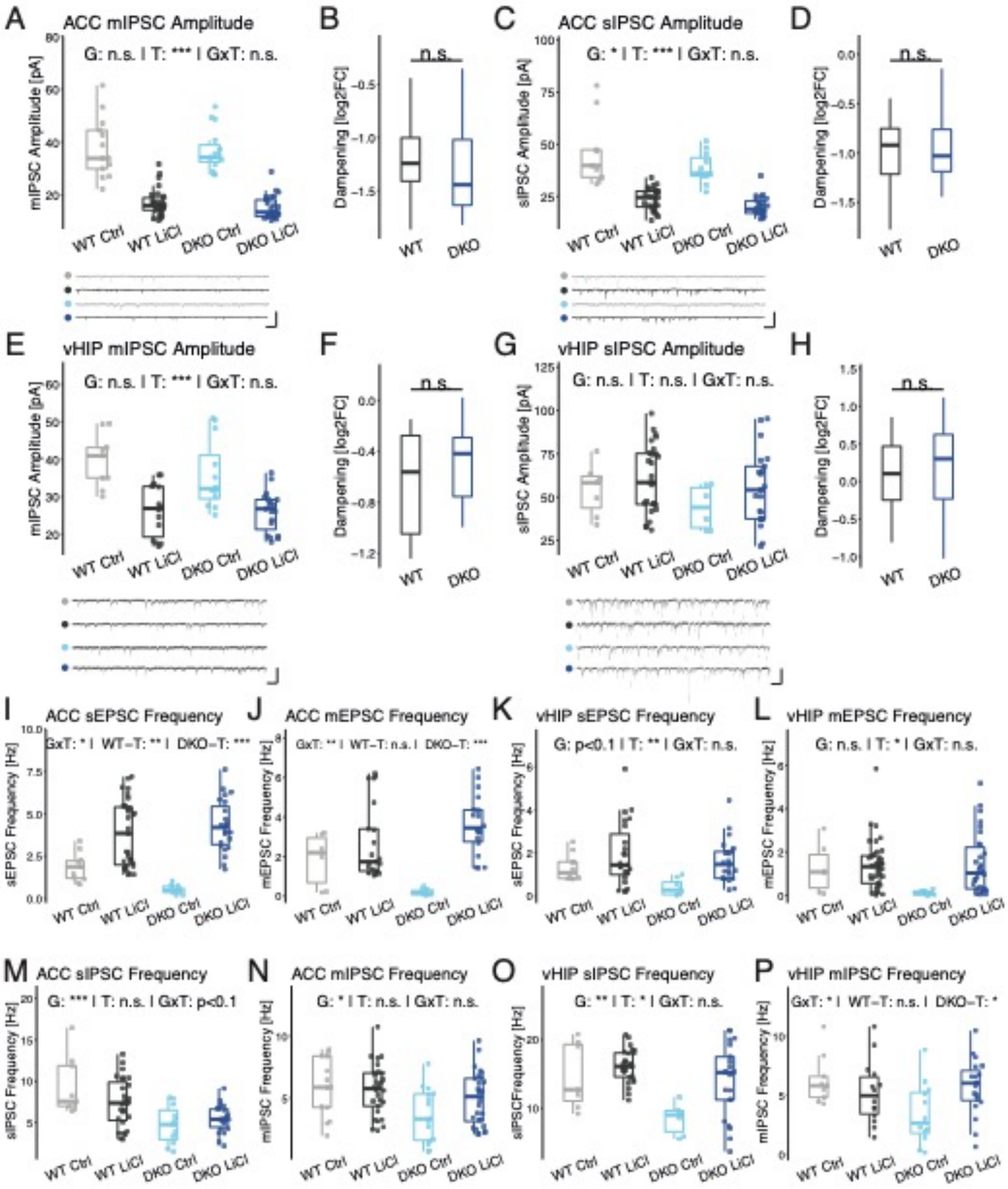

Figure S11. Extended data from whole-cell recordings in ACC and vHIP. Whole-cell voltage-clamp recordings in layer 2/3 pyramidal cells in the anterior cingulate cortex (ACC) area of the medial prefrontal cortex (mPFC) and pyramidal cells in the CA1 region of the ventral hippocampus (vHIP). (A-H) Average miniature and spontaneous inhibitory post-synaptic potential (mIPSC & sIPSC) amplitudes with example traces (scale bars x: 0.2 s & y: 0.1 nA) and the log2-transformed fold change of the respective amplitudes in LiCl compared to Ctrl mice. (I-L) Average miniature and spontaneous excitatory post-synaptic potential (mEPSC & sEPSC) frequency. (M-P) Average mIPSC and sIPSC frequency measured in ACC and vHIP. * P < 0.05; ** P < 0.01; *** P < 0.001; n.s. not significant; P-values refer to univariate two-way ANOVA with Type 2 sum-of-squares; simple effects were tested in a similar but unifactorial ANOVA procedure; WT: wildtype mice; DKO: Bhlhe40/41 double-knockout mice; Ctrl: vehicle control; LiCl: lithium-treated; G: genotype main effect; T: treatment main effect; GxT: interaction effect; simple T: simple effects; DKO-T: DKO simple treatment effect; WT-T: WT simple treatment effect.

bioRxiv preprint doi: https://doi.org/10.1101/2024.07.25.605130; this version posted July 25, 2024. The copyright holder for this preprint (which was not certified by peer review) is the author/funder, who has granted bioRxiv a license to display the preprint in perpetuity. It is made available under aCC-BY 4.0 International license.

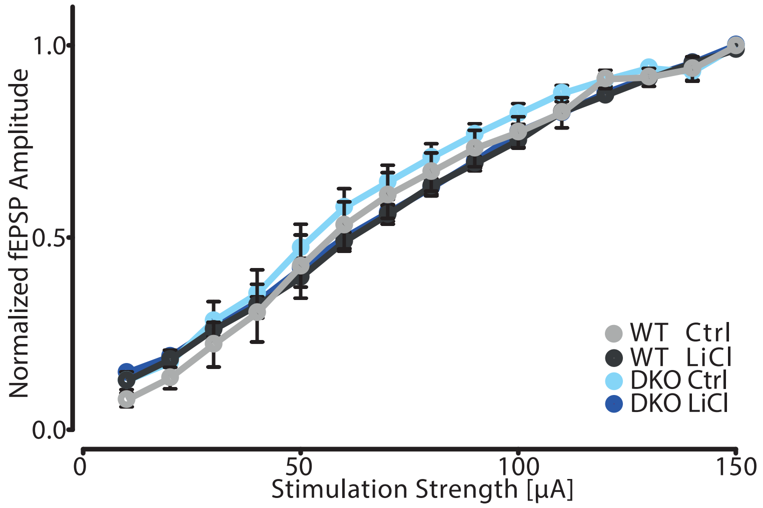

Figure S12. Extended Data on LTP Recordings in the CA1 Region of the Hippocampus. Input output (I/O) relationship of the mean (+/-SEM) normalized field excitatory post-synaptic potential (fEPSP) measured in the CA1 region plotted against the stimulation current injected at the Schaffer collaterals in the stratum radiatum at the CA3/CA1 junction did not show any differences in basic synaptic transmission between groups WT: wildtype mice; DKO: Bhlhe40/41 double-knockout mice; Ctrl: vehicle control; LiCl: lithium-treated

bioRxiv preprint doi: https://doi.org/10.1101/2024.07.25.605130; this version posted July 25, 2024. The copyright holder for this preprint (which was not certified by peer review) is the author/funder, who has granted bioRxiv a license to display the preprint in perpetuity. It is made available under aCC-BY 4.0 International license.

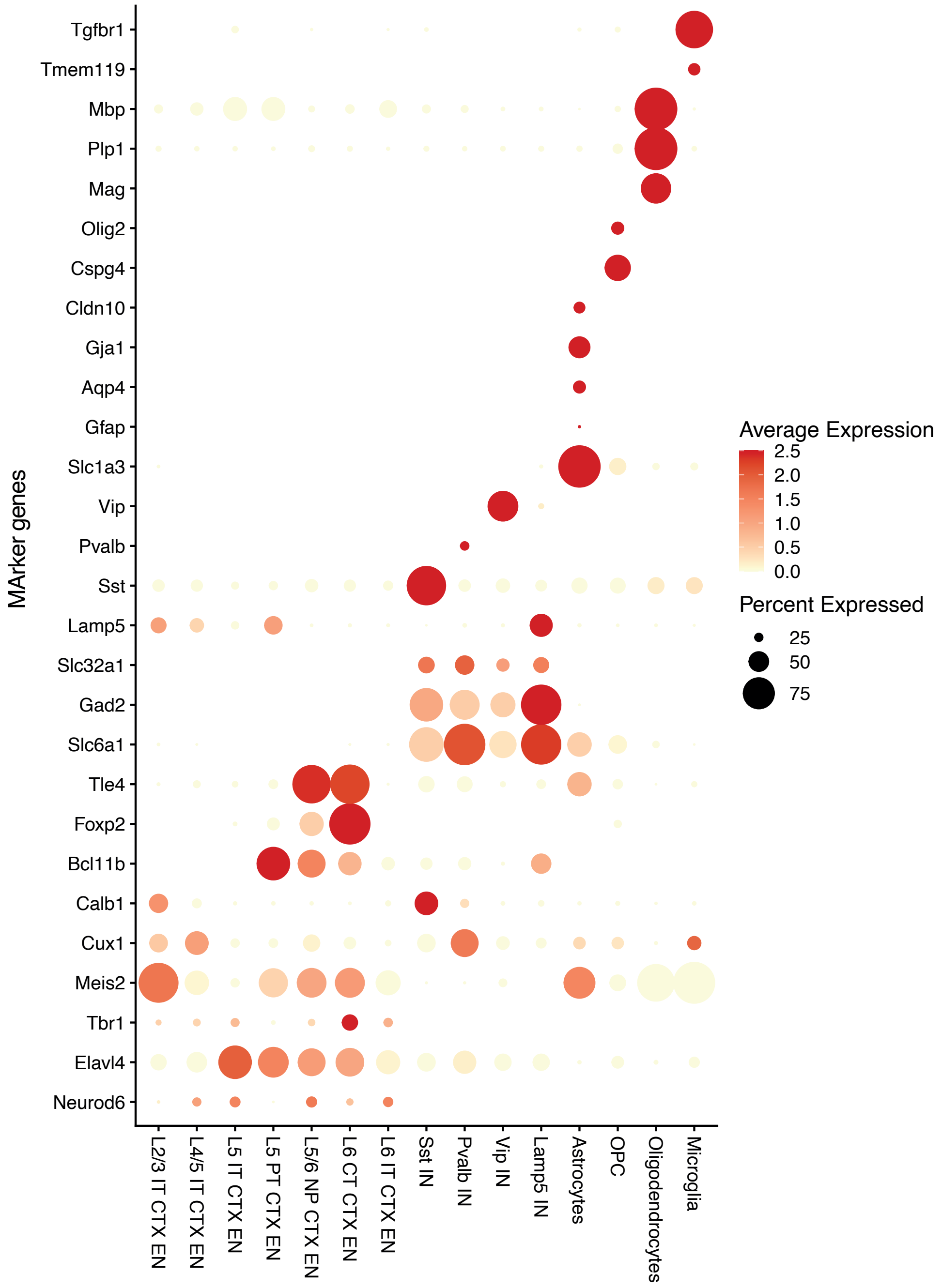

849

850 **Figure S13. Dotplots of selected cluster marker genes**. Normalized average expression is shown in
851 color scale, abundance of expressing cells in percent as dot size.

852

bioRxiv preprint doi: https://doi.org/10.1101/2024.07.25.605130; this version posted July 25, 2024. The copyright holder for this preprint (which was not certified by peer review) is the author/funder, who has granted bioRxiv a license to display the preprint in perpetuity. It is made available under aCC-BY 4.0 International license.

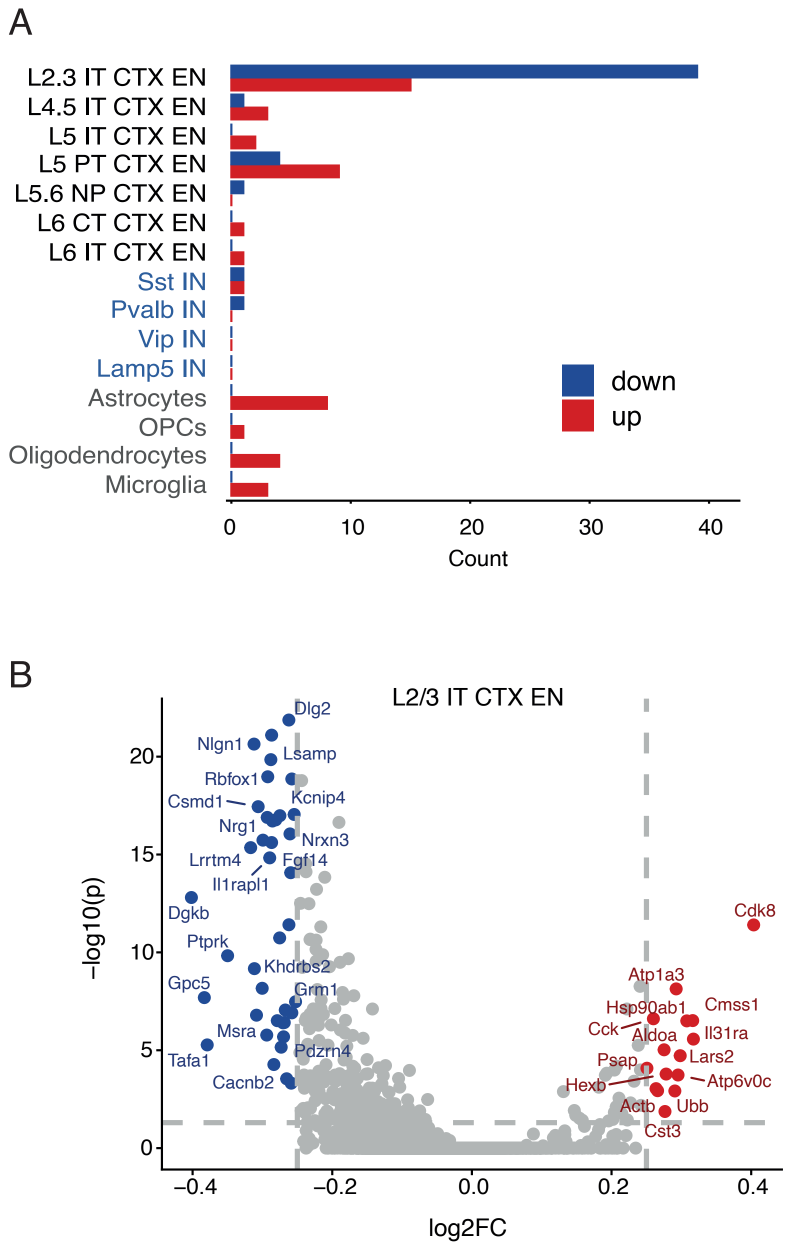

853

854 **Figure S14. Differential gene expression analysis in wt and DKO treated with lithium by single**
855 **cell RNAseq.** (A) Barplot of numbers of significantly deregulated genes between the groups reveals a
856 highly selective response in layer 2/3 excitatory neurons compared to all other cell types. (B) Volcano
857 plot of deregulated genes in layer 2/3 excitatory neurons. On the Y axis the -log transformed adjusted
858 P-value is shown and on the X-axis the average log2-transformed fold-change is plotted at the indicated
859 thresholds (dashed lines).

860

bioRxiv preprint doi: https://doi.org/10.1101/2024.07.25.605130; this version posted July 25, 2024. The copyright holder for this preprint (which was not certified by peer review) is the author/funder, who has granted bioRxiv a license to display the preprint in perpetuity. It is made available under aCC-BY 4.0 International license.

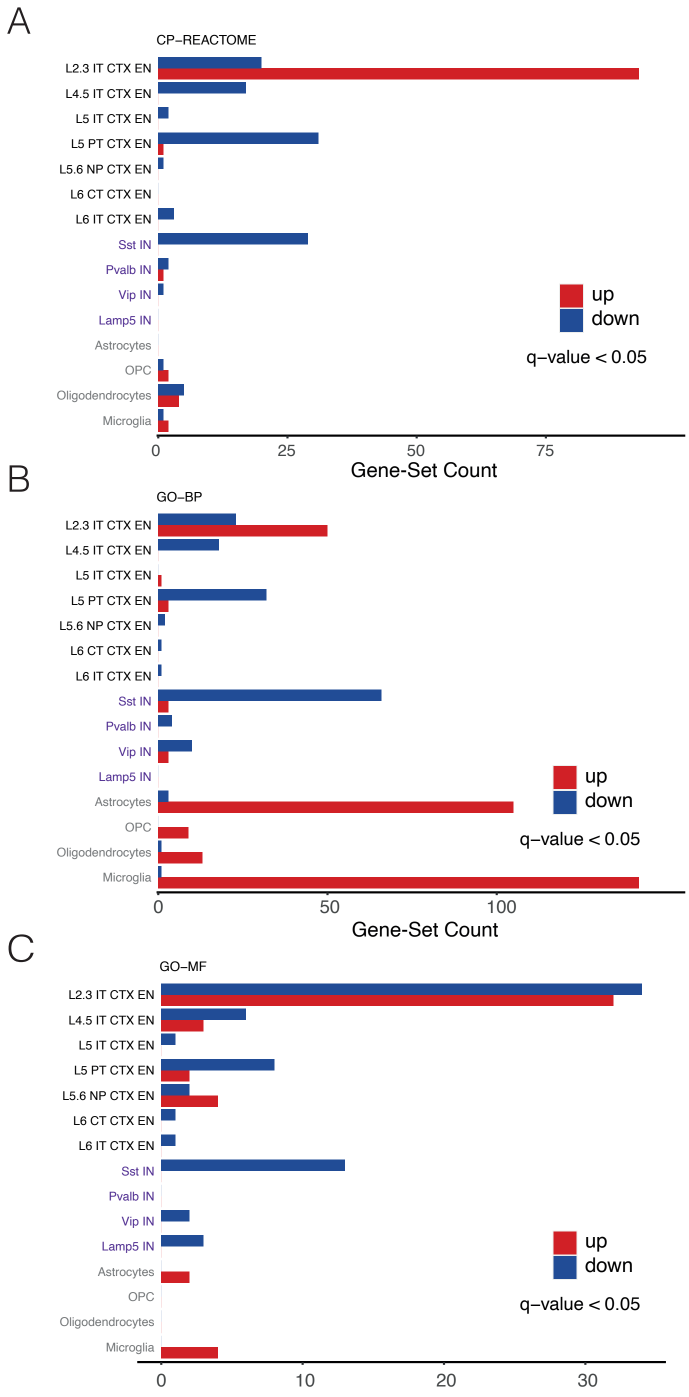

Figure S15. Differentially regulated gene-sets in wt and DKO treated with lithium identified with GSEA. (A-C) Barplot of numbers of significantly deregulated gene-sets (corrected q-val < 0.05) between the groups reveals a prominent response in layer 2/3 excitatory neurons in the pathway collection from reactome (A), gene ontology biological process (GO-BP) (B), and the gene ontology molecular function (GO-MF) (C) subcollections.

bioRxiv preprint doi: https://doi.org/10.1101/2024.07.25.605130; this version posted July 25, 2024. The copyright holder for this preprint (which was not certified by peer review) is the author/funder, who has granted bioRxiv a license to display the preprint in perpetuity. It is made available under aCC-BY 4.0 International license.

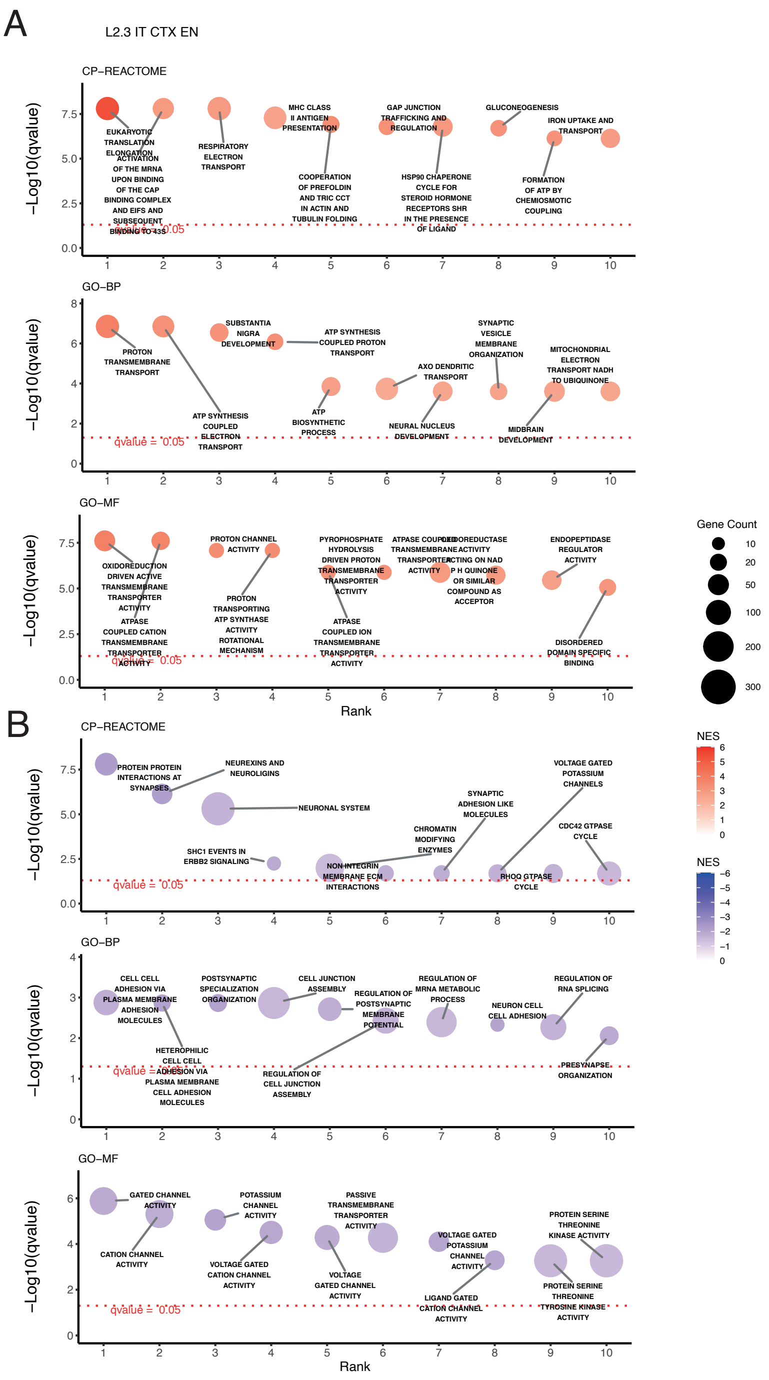

Figure S16. Top deregulated gene-sets in layer 2/3 excitatory neurons from wt and DKO treated with lithium identified with GSEA. (A) Ten most significantly upregulated and (B) downregulated gene-sets from the pathway databases reactome (top), the gene ontology biological process (GO-BP) (middle), and the gene ontology molecular function (GO-MF) (bottom) subcollections. Upregulated gene-sets are mainly associated with metabolism and mitochondrial ATP-synthesis. Down-regulated gene-sets are mainly associated with the synapse and cation-channel activities.

bioRxiv preprint doi: https://doi.org/10.1101/2024.07.25.605130; this version posted July 25, 2024. The copyright holder for this preprint (which was not certified by peer review) is the author/funder, who has granted bioRxiv a license to display the preprint in perpetuity. It is made available under aCC-BY 4.0 International license.

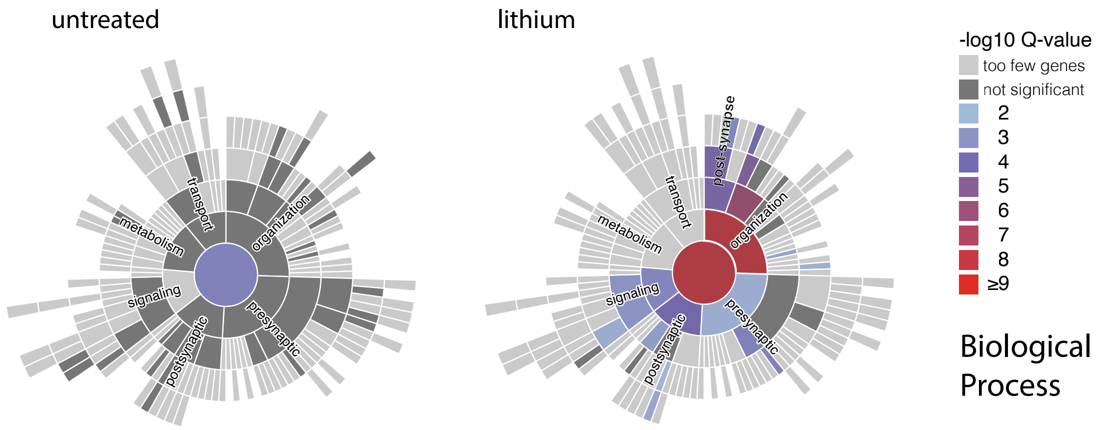

Figure S17. SynGO enriched gene-sets in layer 2/3 excitatory neurons from untreated and lithium treated wt and DKO scRNAseq ACC samples. Enrichment of synaptic gene-sets from the SynGO reveals most significant effects in lithium treated animals in biological processes associated with synaptic organization and post-synaptic functions.
